# Low-Temperature Self-Healing of a Microcapsule-Type Protective Coating

**DOI:** 10.3390/ma10091079

**Published:** 2017-09-14

**Authors:** Dong-Min Kim, Yu-Jin Cho, Ju-Young Choi, Beom-Jun Kim, Seung-Won Jin, Chan-Moon Chung

**Affiliations:** Department of Chemistry, Yonsei University, Wonju 26493, Gangwon-do, Korea; dmkimr@yonsei.ac.kr (D.-M.K.); chyujn904@naver.com (Y.-J.C.); cjy0510@gmail.com (J.-Y.C.); sk754@naver.com (B.-J.K.); jinsw0906@yonsei.ac.kr (S.-W.J.)

**Keywords:** low temperature, self-healing, microcapsule, protective coating

## Abstract

Low-temperature self-healing capabilities are essential for self-healing materials exposed to cold environments. Although low-temperature self-healing concepts have been proposed, there has been no report of a microcapsule-type low-temperature self-healing system wherein the healing ability was demonstrated at low temperature. In this work, low-temperature self-healing of a microcapsule-type protective coating was demonstrated. This system employed silanol-terminated polydimethylsiloxane (STP) as a healing agent and dibutyltin dilaurate (DD) as a catalyst. STP underwent a condensation reaction at −20 °C in the presence of DD to give a viscoelastic product. The reaction behavior of STP and the viscoelasticity of the reaction product were investigated. STP and DD were separately microencapsulated by in situ polymerization and interfacial polymerization methods, respectively. The STP- and DD-loaded microcapsules were mixed into a commercial enamel paint, and the resulting formulation was applied to glass slides, steel panels, and mortars to prepare self-healing coatings. When the self-healing coatings were damaged at a low temperature (−20 °C), STP and DD were released from broken microcapsules and filled the damaged area. This process was effectively visualized using a fluorescent dye. The self-healing coatings were scratched and subjected to corrosion tests, electrochemical tests, and saline solution permeability tests. The temperature of the self-healing coatings was maintained at −20 °C before and after scratching and during the tests. We successfully demonstrated that the STP/DD-based coating system has good low-temperature self-healing capability.

## 1. Introduction

Self-healing materials are artificial materials that can self-heal after being damaged. Recently, self-healing materials have attracted much attention because they can extend material lifetime, reduce maintenance cost, and enhance public safety [1,2,3,4,5,6]. Self-healing materials can be classified broadly into two groups: intrinsic and extrinsic [5]. Intrinsic-type materials possess a latent self-healing functionality that is triggered by damage or by an external stimulus, so they can heal damage by the materials themselves. In extrinsic-type self-healing materials, the damage is recovered by the release of healing agent from microcontainers embedded in the matrix. The extrinsic materials can be generally categorized into two main groups: capsule based and vascular. For capsule-based systems, when the capsules are ruptured by damage, the healing agent is released from the ruptured capsules, and fills the damaged region which then undergoes a healing reaction. A vascular type works in a similar way, but the capsules are replaced by a vascular network in one, two or three dimensions. Compared to the intrinsic system, the extrinsic system has a major advantage in healing larger damage volume [6].

Although good self-healing performances have been demonstrated, most reported self-healing materials show their self-healing capability at room temperature or higher [1,2,3,4,5,6,7,8,9,10,11,12,13,14,15,16,17,18]. However, low-temperature self-healing capability is essential for self-healing materials exposed to cold environments. For example, most self-healing materials for concrete or metals can be exposed to a low-temperature environment in winter. For aerospace applications, self-healing materials must have a self-healing function at temperatures as low as −60 °C. Only a few reports on low-temperature self-healing have been based on intrinsic-type materials. Kalista et al. probed the self-healing of ballistic puncture of poly(ethylene-*co*-methacrylic acid) films by the energy transferred to the films from projectiles, and tested the self-healing response in ballistic impact tests at −50 °C [19]. Self-healing at temperatures as low as −20 °C was reported by Li et al. using a poly(dimethylsiloxane) crosslinked by coordination complexes [20].

To the best of our knowledge, there has been no report on a capsule-based low-temperature self-healing system wherein the healing ability was demonstrated at low temperature. Yuan et al. reported a low-temperature self-healing system utilizing microencapsulated epoxy and a mercaptan hardener as a two-component healing agent [21]. In their work, specimens were stored at temperatures as low as −10 °C before and after damage creation, but the self-healing performance of the system was evaluated at room temperature. As they state in the paper, their test method cannot completely rule out the possibility that self-healing might occur as the specimen temperature increased to room temperature. Raimondo et al. studied the polymerization of a 5-ethylidene-2-norbornene/dicyclopentadiene healing agent at −50 °C for aerospace applications, but they conducted self-healing tests at room temperature [22]. Hillewaere et al. reported self-healing of epoxy thermosets with thiol-isocyanate chemistry at −2 °C, but the self-healing performance was studied at room temperature [23]. On the other hand, Wang et al. designed and fabricated a fiber-reinforced composite system having high healing efficiency at around −50 °C [24]. This system was composed of three-dimensional hollow vessels that deliver and release healing agents and a conductive element to provide heat to defrost and promote healing reactions. Therefore, the development of a low-temperature self-healing system that not only can effectively show self-healing capability in low-temperature tests but also can work without an additional heating element is a remaining challenge in the field of extrinsic-type self-healing materials.

In this paper, we report a microcapsule-type self-healing protective coating that can self-heal at temperatures as low as −20 °C. This coating system is the first example of an extrinsic-type low-temperature self-healing system wherein the healing ability was demonstrated at low temperature without the use of a heating element. Silanol-terminated polydimethylsiloxane (STP) and dibutyltin dilaurate (DD) were used as a healing agent and a catalyst, respectively, and they were separately microencapsulated to prepare a dual-capsule self-healing coating. The reaction behavior of STP in the presence of DD and the release of STP and DD from ruptured microcapsules were studied at −20 °C. We evaluated the self-healing performance of the coating at −20 °C using corrosion tests, electrochemical tests, and saline solution permeability tests.

## 2. Materials and Methods

### 2.1. Materials

**Figure 1:**
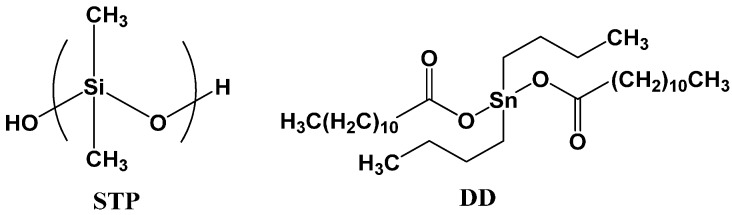
Chemical structures of silanol-terminated polydimethylsiloxane (STP) as a healing agent and dibutyltin dilaurate (DD) as a catalyst.

Urea, a formaldehyde solution (37 wt %), poly(ethylene-alt-maleic anhydride) (EMA), resorcinol, 1-octanol, ammonium chloride, tolylene-2,4-diisocyanate (TDI), 1,4-butanediol (BD), ethylene glycol (EG), gum arabic, cyclohexanone, and chlorobenzene were purchased from Sigma-Aldrich (St. Louis, MO, USA) or Tokyo Chemical Industry (Nihonbashi, Japan) (TCI) for use in the microencapsulation process. Gum Arabic and EMA were used as surfactants. Silanol-terminated polydimethylsiloxane (STP) (DMS-S12) and dibutyltin dilaurate (DD) were purchased from Gelest (Morrisville, PA, USA) and Sigma-Aldrich (St. Louis, MO, USA), respectively, and were used as core materials (Figure 1). *n*-Hexadecane (melting point = 18 °C) was purchased from Sigma-Aldrich and was used as a comparison core material. A fluorescent fluid (OIL-GLO 44-P) consisting of polyol ester oil, mineral oil, petroleum hydrocarbon, perylene dye and naphthalimide dye was purchased from Spectronics (Westbury, NY, USA). An enamel paint (KCI 7200, white) was purchased from Kunsul Chemical Industrial Co. (Seoul, Korea). Steel panels (20 mm × 50 mm × 1 mm) were used in the corrosion test and the electrochemical test. Mortar specimens (40 mm × 40 mm × 130 mm) were prepared with cement, sand and water with a mass ratio of 2:6:1, respectively, according to a KSF2476 standard method. Mortar paste was first cured in a mold for 48 h at room temperature. Each mortar was further cured for 5 days in water and then finally cured for 7 days under ambient conditions.

### 2.2. Instruments

A low-temperature chamber (LTC-27, Lab house, Pocheon, Korea) was used to study the reaction of STP and to evaluate self-healing performance. A mechanical stirrer (NZ-1000, Eyela, Tokyo, Japan) equipped with a propeller-type impeller was used for microencapsulation. An Advanced Rheometric Expansion System (ARES, Rheometric Scientific, Piscataway, NJ, USA) was used to examine the viscoelasticity of the reacted STP. A fluorescence microscope (BX-51, Olympus, Tokyo, Japan) was used to take pictures of microcapsules and scratches on the surfaces of the coatings. Microcapsule size was analyzed using a CCD camera (HK6U3Cool, Koptic, Seoul, Korea) attached to the microscope and image analysis software (HKBasic, Koptic). An electronic balance (ML303, Mettler Toledo, Columbus, OH, USA) was used to weigh materials used in this work. An infrared thermometer (35639-20, Oakion, Vernon Hills, IL, USA) was used to measure the temperature of coating samples and the aqueous electrolyte. Infrared (IR) spectra were recorded on a Fourier transform infrared (FT-IR) spectrophotometer (Spectrum One B, Perkin Elmer Co., Waltham, MA, USA). A field emission scanning electron microscope (FE-SEM) (SU-70, Hitachi, Tokyo, Japan) was used to examine the morphology of the microcapsules. Thermogravimetric analysis (TGA) was conducted using a Shimadzu TA-50 to investigate the core composition of DD microcapsules. A potentiostat/galvanostat (273A, Ametek, Berwyn, PA, USA) was used to examine the conductivity of the coated substrate.

### 2.3. Reaction Conversion Measurement

**Figure 2:**
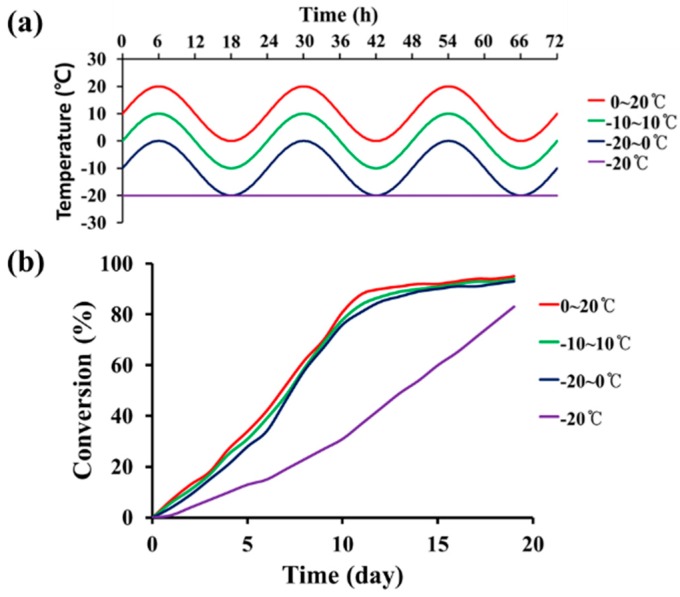
(**a**) Temperature conditions for STP/DD reaction; (**b**) Reaction conversion of silanol groups versus time at each temperature condition.

STP and DD were mixed at a mass ratio of 10:1, and the mixture was poured in molds that were 25 mm in diameter and 1 mm in height. The mixture was allowed to react for 19 days at −20 °C (constant temperature), −20–0 °C (cycle), −10–10 °C (cycle), and 0–20 °C (cycle) (Figure 2a). Samples were taken periodically and analyzed by FT-IR spectroscopy. The mass ratio of 10:1 was selected because viscoelastic products were obtained from STP and DD mixtures with a mass ratio range of 8:1–12:1 in preliminary experiments. The degree of conversion of silanol groups of STP was analyzed by an IR band-ratio method. The absorbance of the O-H stretch band at 3330 cm^−1^ was divided by that of a reference C-H stretch band at 2960 cm^−1^ (Figure S1). The degree of conversion was calculated using the following equation,

Conversion (%) = [1 − (A_3330_/A_2960_)_after reaction_/(A_3330_/A_2960_)_before reaction_] × 100 (1)

where A_3330_ and A_2960_ are the absorbance values of the 3330 and 2960 cm^−1^ absorption bands of STP, respectively.

### 2.4. Viscoelasticity Measurement

STP and DD were mixed at a mass ratio of 10:1, and the mixture was poured into molds that were 25 mm diameter and 1 mm height and allowed to react for 19 days at −20 °C. Disk-like samples of the product were separated from the molds. The viscoelasticity of the reacted STP was examined using an ARES rheometer (Rheometric Scientific, Piscataway, NJ, USA) at −20, 5, and 30 °C. This experiment was conducted under constant strain and temperature, while frequency was varied from 0.1 to 510.0 rad/s. Then, the experimental results were plotted in terms of G’ and G” versus frequency.

### 2.5. Microencapsulation of STP and n-Hexadecane

Water (20 mL) and a 2.5 wt % aqueous solution of EMA (5 mL) were added to a 100-mL beaker. The beaker was placed in a water bath equipped with a mechanical stirrer. Urea (0.503 g), ammonium chloride (0.050 g), and resorcinol (0.050 g) were added under agitation at 300 rpm. The pH of the resulting mixture was adjusted to 3.5 using a 10 wt % NaOH solution. The mixture was stirred at 1000 rpm, and two drops of 1-octanol were added. Then, 8 mL of STP were added, and the mixture was stirred at 1000 rpm for 15 min to form a stable emulsion. After adding a 37 wt % formaldehyde solution (1.456 g), the temperature of the mixture was increased to 60 °C, which was maintained for 4.5 h. The resulting suspension was cooled to room temperature, and microcapsules were filtered using vacuum filtration. The microcapsules were washed with water and ethyl alcohol and air-dried for 48 h (yield = 91%). *n*-Hexadecane was microencapsulated in a similar fashion. Measurement of microcapsule payload was conducted using an electronic balance. After weighing, the STP-loaded microcapsules were crushed by pressing them in a 5-mL vial and were washed twice with ethyl alcohol to remove the core (STP). The remaining shell material was dried under ambient conditions and weighed.

### 2.6. Microencapsulation of DD

TDI (11.50 g, 0.066 mol) was dissolved in cyclohexanone (70 mL) under nitrogen in a 150-mL round-bottom flask. The flask was placed in an oil bath, and BD (2.70 g, 0.030 mol) was added at 55 °C. The resulting solution was heat at 80 °C for 24 h. Then, the reaction mixture was heated under vacuum at 80 °C for 4 h and then at 100 °C for 1 h to remove the solvent and unreacted reagents and to obtain a prepolymer. Gum arabic (3.10 g) was dissolved in distilled water (20 mL) in a 100-mL beaker. The prepolymer (3.00 g) was dissolved in chlorobenzene (32.00 g), and DD (1.00 g) was then added. The prepolymer/DD solution was slowly poured into the gum arabic solution to give an emulsion. EG (3.00 g) as a chain extender was slowly added to the emulsion at 50 °C. The mixture was heated; when the temperature reached 70 °C, the reaction was stopped. After filtration, the microcapsules were washed with distilled water and air-dried for 72 h (yield = 84%). Measurement of the composition of DD-loaded microcapsules was conducted using the TGA instrument and the electronic balance. After weighing, the DD microcapsules were crushed and subjected to isothermal TGA at 25 °C to measure the content of chlorobenzene in the microcapsules (Figure S2). Chlorobenzene readily evaporated at 25 °C within 10 min, and DD and shell material remained. On the other hand, the DD-containing microcapsules were crushed by pressing them in a 5-mL vial and washing twice with ethyl alcohol to remove the core. The remaining shell material was dried under ambient conditions and weighed.

### 2.7. Microencapsulation of Fluorescent Core

Microcapsules containing fluorescent core were prepared and used to visualize the release of the core materials from broken microcapsules. Fluorescent cores were prepared by adding the fluorescent fluid into STP, DD or *n*-hexadecane. The mass ratios of the components of the fluorescent cores were as follows: STP/fluorescent fluid = 9:1, chlorobenzene/DD/fluorescent fluid = 32:0.9:0.1, and *n*-hexadecane/fluorescent fluid = 9:1. The procedure of microencapsulation of the fluorescent core containing DD was the same as that of DD encapsulation described above. The procedure of microencapsulation of the fluorescent core containing STP or *n*-hexadecane was the same as that of the STP microencapsulation described above.

### 2.8. Preparation of the Coating Samples

The STP- and DD-loaded microcapsules were uniformly dispersed into the commercial enamel paint to prepare self-healing coatings. The mass ratio of STP capsules/DD capsules/enamel paint was 18:7:75. The mass ratio of STP capsules/DD capsules of 18:7 corresponds to the 10:1 mass ratio of STP and DD. The *n*-hexadecane-containing microcapsules were mixed into the enamel paint with a mass ratio of *n*-hexadecane capsules/enamel paint = 25:75 to prepare comparison coatings. The fluorescent core-containing microcapsules were mixed into the enamel paint with a mass ratio of microcapsules/enamel paint = 25:75. The resulting coating formulation was applied to the surfaces of glass slides for release testing, steel panels for corrosion and electrochemical testing, and mortar specimens for saline solution permeability testing. The steel panels were washed with ethyl alcohol and dried under ambient conditions before coating. The coated samples were dried for 2–3 days at room temperature. Control coating samples were prepared in a similar fashion without adding microcapsules. Coating thicknesses were about 550 μm (on slide glass) and about 90 μm (on the steel panel and mortar). When thick coatings (>500 μm) formed on the steel panel or mortar, the original scratch width was not maintained and tended to decrease to some extent. It was expected that the exact evaluation of self-healing performance would not be possible using the thick coatings on steel panel and mortar, so relatively thin coatings (<100 μm) were prepared.

### 2.9. Release Test

The coating formulation containing fluorescent-core microcapsules was applied to the surfaces of glass slides and dried. The coated glass slides were stored at −20 °C for 24 h in the low-temperature chamber. Immediately after opening the chamber door, a scratch was generated in each coating with a razor blade. It was confirmed that each cut was deep enough to reach the surface of the glass slide. Optical microscopy showed that the scratch width was about 20 μm. Scratching was performed inside the chamber within 30 s to avoid an increase in sample temperature. The scratched samples were left in the chamber at −20 °C for 10–15 min. The scratched coatings were observed using a fluorescence microscope within 1–2 min immediately after taking the samples out of the chamber. During the observation, the temperature of the coating samples increased from −20 to −17 °C.

### 2.10. Corrosion Test

The STP/DD-based self-healing coatings and control coatings were applied to one side of the steel panels. The other side of each panel was covered with enamel paint. The coated steel panels were stored at −20 °C for 24 h in the low-temperature chamber. Immediately after opening the chamber door, cross scratches were applied to the self-healing and control coatings with a razor blade inside the chamber. It was confirmed that each cut was deep enough to reach the surface of the steel panel. Optical microscopy showed that the scratch width was about 20 μm. The scratching was performed inside the chamber within 30 s to avoid an increase in sample temperature. After the scratched coatings were left at −20 °C for 12 h in the chamber, they were immediately immersed in a 25 wt % NaCl aqueous solution that was maintained at −20 °C in the chamber as an accelerated corrosion test. After 48 h, the coatings were washed with distilled water and wiped to remove residual water, and the coating surfaces were observed by optical microscopy. For each kind of sample, at least three specimens were used for the corrosion test.

### 2.11. Electrochemical Test

The STP/DD-based self-healing coatings and control coatings were applied to one side of the steel panels. The coated steel panels were stored at −20 °C for 24 h in the chamber. Immediately after opening the chamber door, cross scratches were applied to the self-healing and control coatings with a razor blade inside the chamber. It was confirmed that each cut was deep enough to reach the surface of the steel panel. Optical microscopy showed that the scratch width was about 20 μm. The scratching was performed inside the chamber within 30 s to avoid an increase in sample temperature. The scratched coatings were left at −20 °C for 12 h in the chamber. Each electrochemical cell was fabricated immediately after taking out a scratched coating sample from the chamber. The fabrication took less than 1 min. Electrochemical tests were conducted using a computer-controlled potentiostat/galvanostat in a conventional three-electrode electrochemical cell equipped with a platinum counter electrode, an Ag/AgCl electrode in a saturated NaCl aqueous solution as the reference electrode, and the coated steel panel as the working electrode (Figure S3). During the test, dry ice was used to keep the coated steel panel and electrolyte at −20 °C. Steady-state conduction was measured between the coated steel panel and a platinum electrode held at −2 V through an aqueous electrolyte (0.1 M Na_2_SO_4_). The current passing through the specimen was recorded using Electrochemistry PowerSuite software. At least three specimens were used for each kind of sample, and an average of the measured values was calculated.

### 2.12. Saline Solution Permeability Test

The self-healing coatings and control coatings were applied to one rectangular side of the square column mortars (Figure S4). Four side surfaces adjacent to the coated side were covered with epoxy resin. The coated mortar specimens were stored at −20 °C for 24 h in the low-temperature chamber. Immediately after opening the chamber door, scratches were applied to the self-healing and control coatings with a razor blade inside the chamber. It was confirmed that each cut was deep enough to reach the surface of the mortar. Optical microscopy showed that the scratch width was about 25 μm. After the scratched coatings were weighed, they were left at −20 °C for 12 h in the chamber. The scratching and weighing took only about 30 s, and the temperature of the samples was maintained at −20 °C. The scratched surface was immediately immersed in a saline solution (25 wt % NaCl aqueous solution) that was being maintained at −20 °C in the chamber. After 48 h, the increase in the mass of mortar due to the absorbed saline solution was determined by weighing each specimen. For each kind of sample, three replicates were tested, and an average of the measured mass was calculated.

## 3. Results and Discussion

### 3.1. Healing Agent

In this study, STP and DD were used as a healing agent and a catalyst, respectively (Figure 1). Healing agents used in low-temperature self-healing must have several specific properties. First, they should have sufficient release property at low temperatures because they need to flow out of broken capsules and fill damaged region. STP has a melting point of −60 °C and a high release property above −60 °C. Although DD has a melting point of 23 °C, it was dissolved in chlorobenzene for use as the core in microencapsulation, and the solution can be released at low temperature. The release properties of the two compounds is discussed further below. Other reasons for the selection of STP and DD include the good water repellent property of STP and the low environmental toxicity of DD.

Secondly, the low-temperature self-healing agent needs to be cured at low temperature after filling the damaged region. STP undergoes a condensation reaction of silanol end groups in the presence of DD at room temperature [25]. In this study, we investigated the low–temperature condensation reaction of STP (Figure 2). As shown in Figure 2a, the reaction of STP was examined during 24-h temperature cycles or at a constant temperature. The temperature cycles were selected based on the environmental temperature cycles of Cheolwon, which is the coldest area in South Korea in the winter. The selected temperature ranges in this work were −20 °C (constant temperature), −20–0 °C (cycle), −10–10 °C (cycle), and 0–20 °C (cycle). The reaction of STP was studied using a STP/DD 10:1 (by mass) mixture, and the conversion of silanol groups was measured by FT-IR (Figure 2b and Table 1). The reaction rates were similar when the reactions were conducted under the three temperature cycles: the conversion reached about 80% after 10 days and increased further at slower rates. When the reaction was performed at a constant temperature of −20 °C, the conversion increased slowly, reaching 83% after 19 days. The viscosity of STP increased from 0.04 dL/g to 1.08–1.30 dL/g due to the reaction (Table 1), implying that the molecular weight of STP greatly increased by the end of the group reactions. We found that the reaction time of the mixture greatly depended on the amount of the mixture. When a small amount (approximately 0.01 mL) of STP/DD 10:1 mixture was tested, it took only 12 h for the mixture to reach 68% conversion at −20 °C. Thus, 12 h was enough time for self-healing in this study because small amounts of STP and DD could be released from ruptured microcapsules and fill the damaged region.

**Figure 3:**
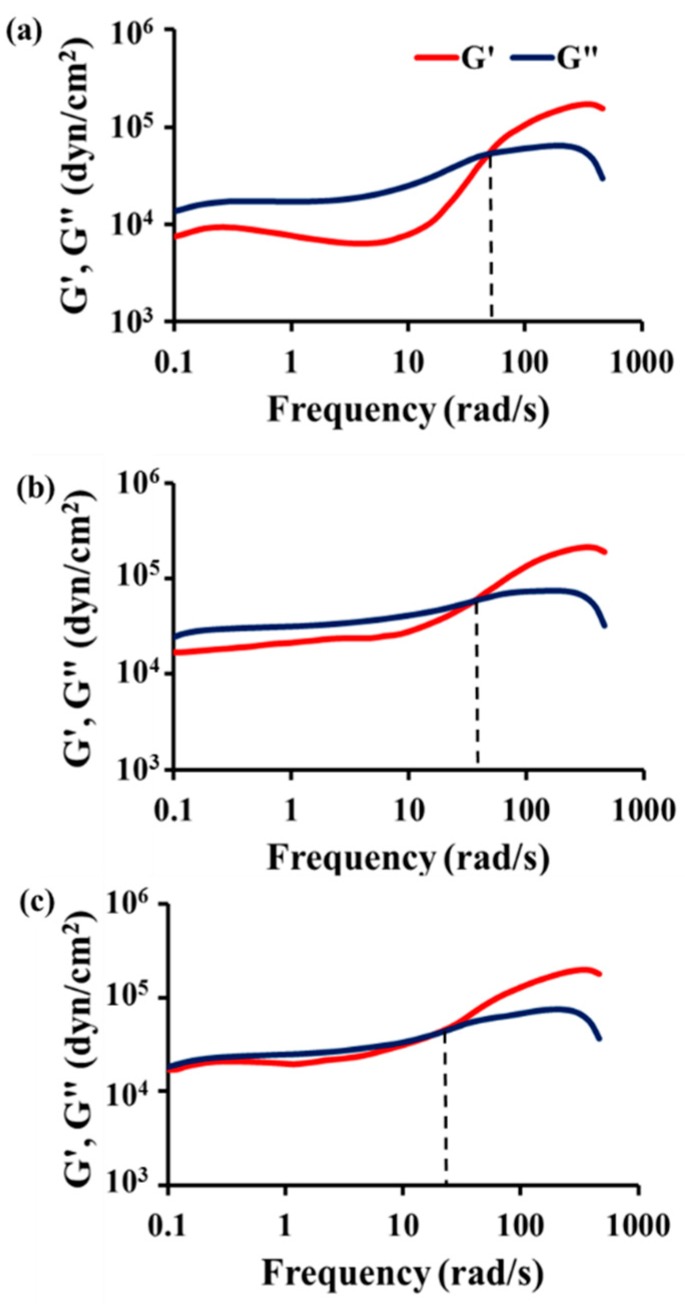
Storage modulus (G’) and loss modulus (G”) of the reaction product measured at (**a**) 30 °C; (**b**) 5 °C; and (**c**) −20 °C. The reaction product was obtained by the reaction of the STP/DD 10:1 (by mass) mixture at −20 °C for 19 days.

Thirdly, the reaction product of the healing agent must display viscoelasticity at low temperature. A viscoelastic material has both viscous and elastic properties [26]. The viscous nature of the product can improve adhesion between the product and coating matrix, resulting in effective sealing of the damaged area. In addition, the viscoelasticity of the reacted healing agent can help the healed region withstand external mechanical stimuli such as traffic-induced vibration at low temperature [25]. If the product is a hard solid without viscoelasticity, secondary cracks would occur in the boundary between the product and coating matrix or inside the product, especially upon exposure to external severe vibration at low temperature. To investigate the viscoelasticity of the reaction product of STP, an STP/DD 10:1 mixture was allowed to react at −20 °C in the low-temperature chamber. We conducted viscoelasticity measurements of the reaction product under constant strain at −20, 5, or 30 °C. Figure 3 shows the data of storage modulus (G’) and loss modulus (G”) as a function of angular frequency for the reaction product. G’ and G” come close to each other below the crossover point with decreasing measurement temperature, and the crossover points are observed at 19, 36, and 50 rad/s at −20, 5 and 30 °C, respectively. These results indicate that the elasticity of the reaction product increases with decreasing temperature, but the product still shows viscoelasticity at temperatures as low as −20 °C.

### 3.2. Preparation of Self-Healing Coatings

**Figure 4:**
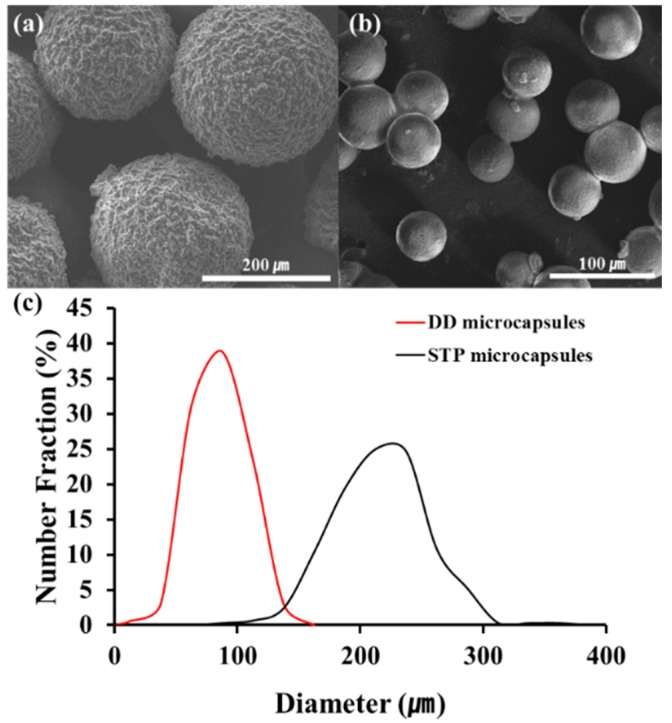
SEM images of (**a**) the STP-containing microcapsules and (**b**) the DD-containing microcapsules; (**c**) Size distribution of STP and DD microcapsules prepared at 1000 rpm.

STP and DD were microencapsulated using urea-formaldehyde polymer and polyurethane as shell materials, respectively. The STP-loaded microcapsules were obtained by in situ polymerization, and the DD-loaded microcapsules were formed by interfacial polymerization. The size and the shape of the microcapsules were observed by scanning electron microscopy (SEM) (Figure 4a,b). Spherical microcapsules were formed at an agitation rate of 1000 rpm. The size distribution of the microcapsules was investigated by optical microscopy (Figure 4c). The average diameters of STP- and DD-loaded microcapsules were 240 and 90 μm, respectively.

Microcapsule composition was studied: the STP-containing microcapsules had a core/shell mass ratio of 71:29, and the DD-containing microcapsules had a chlorobenzene/DD/shell mass ratio of 67:18:15. The microcapsules were dispersed into a commercial enamel paint to prepare a self-healing coating formulation. The coating formulation had a mass ratio of STP capsules/DD capsules/enamel paint of 18:7:75, and an 18:7 ratio of STP capsules/DD capsules corresponds to a 10:1 mass ratio of STP/DD.

### 3.3. Low-Temperature Release of Healing Agent

**Figure 5:**
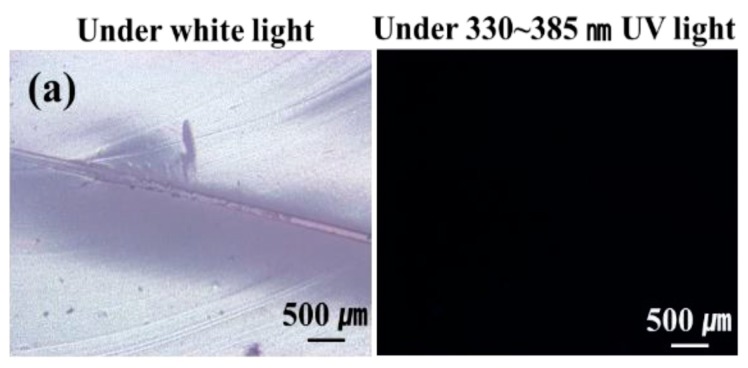

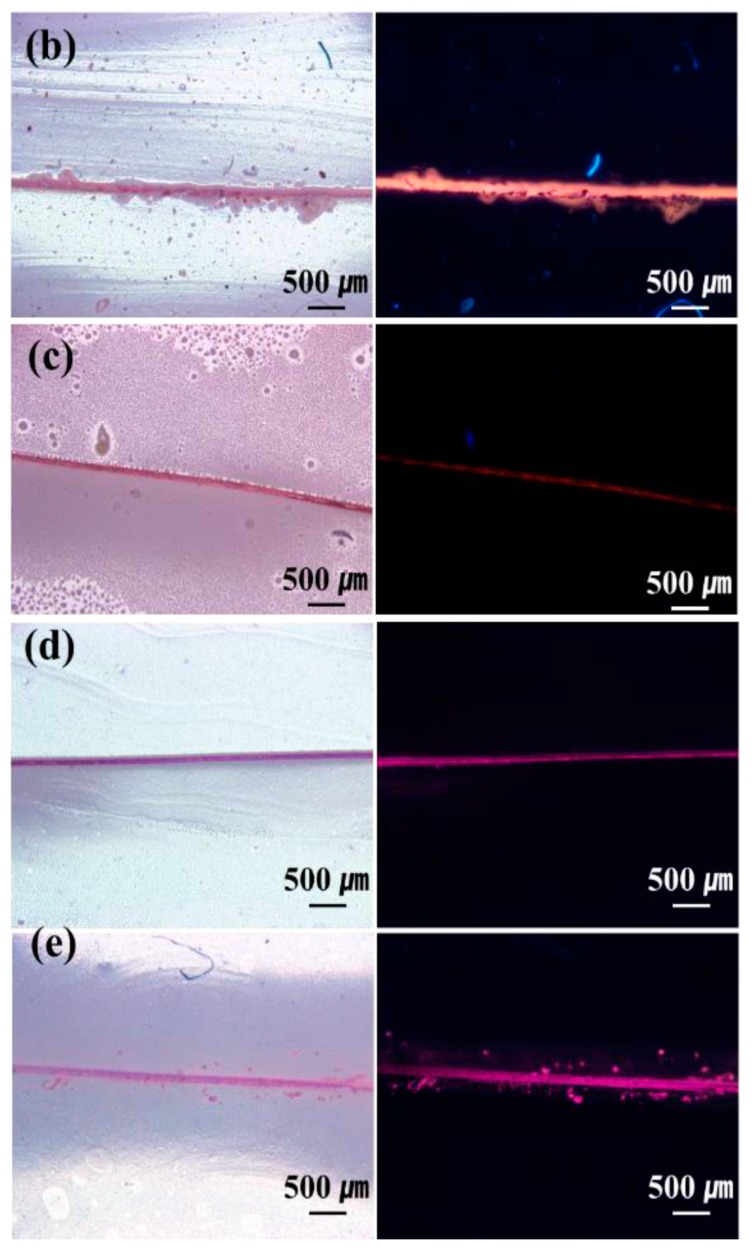
Optical micrographs of scratched regions in the comparison coatings and self-healing coatings under white light and UV light (a fluorescent fluid was mixed in the cores to visualize the release of the core materials upon microcapsule rupture); (**a**) *n*-Hexadecane-based comparison coating observed at −20 to −17 °C; (**b**) *n*-hexadecane-based comparison coating at room temperature (23 °C); (**c**) STP-based coating at −20 to −17 °C; (**d**) DD-based coating at −20 to −17 °C; and (**e**) STP and DD-based self-healing coating at −20 to −17 °C.

The low-temperature release of STP and DD in the self-healing coatings was studied. A fluorescent fluid was mixed with STP or DD to visualize the release of the core materials from broken microcapsules. For comparison, microcapsules containing a mixture of *n*-hexadecane and the fluorescent fluid were prepared and embedded in the enamel paint. After storing and scratching the self-healing coatings and comparison coatings at −20 °C, they were observed by optical microscopy under white light or 330–385-nm UV light at room temperature or lower. Optical microscopy showed that the scratch width was about 20 μm. When the *n*-hexadecane-based comparison coating was observed at −20 to −17 °C, the scratched region was unfilled (Figure 5a). This indicates that *n*-hexadecane did not flow out of the ruptured microcapsules because its melting point (18 °C) is higher than the test temperature. However, when the scratching and observation were conducted at room temperature, which is higher than its melting point, *n*-hexadecane readily flowed out of the microcapsules and filled the damaged region (Figure 5b). When STP- or DD-based coatings were observed at −20 to −17 °C, the scratched region was filled (Figure 5c,d). The STP/DD-based coating showed similar results (Figure 5e). These results imply that STP and DD were readily released from ruptured microcapsules and filled the damaged region when the damage occurred to our self-healing coating at temperatures as low as −20 °C.

### 3.4. Corrosion Test

**Figure 6:**
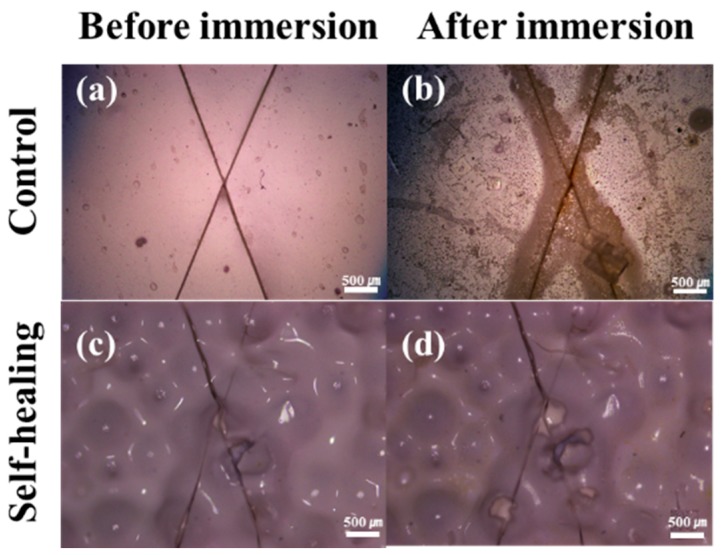
Optical microscope images of scratched control and self-healing coating samples before and after immersion for 48 h in a 25 wt % sodium chloride aqueous solution at −20 °C. (**a**) Control coating before immersion; (**b**) control coating after immersion; (**c**) self-healing coating before immersion; (**d**) self-healing coating after immersion.

The low-temperature self-healing capability of the STP/DD-based self-healing coating system was evaluated through steel corrosion tests (Figure 6). The self-healing coating formulation was applied to steel panels, and the control coating sample was also prepared without microcapsules. After storing the coating samples at −20 °C, damage was induced by scratching the coatings at −20 °C inside the low-temperature chamber (Figure 6a,c). The scratched STP/DD-based self-healing coatings were left at −20 °C for 12 h to induce the reaction of the released healing agent. The scratched and healed self-healing coatings and control coatings were immersed in a 25 wt % aqueous NaCl solution for 48 h at −20 °C in the chamber. All control samples corroded (Figure 6b), but STP/DD-based self-healing coatings showed no visual evidence of corrosion (Figure 6d). Repeated corrosion tests demonstrated good reproducibility.

### 3.5. Electrochemical Test

**Figure 7:**
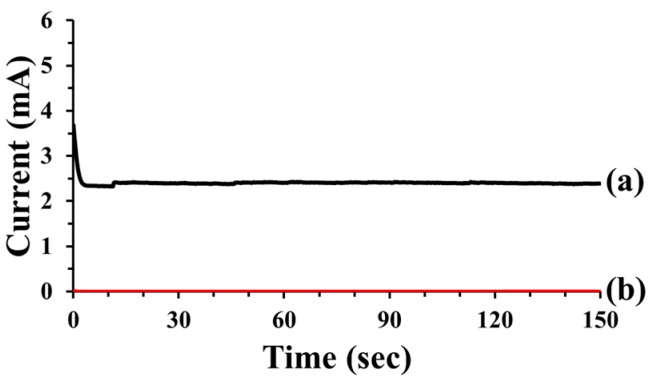
Current versus time for scratched control and self-healing coatings: (**a**) control coating; and (**b**) STP/DD-microcapsule-containing self-healing coating. After preparation of the coatings, scratching, healing and electrochemical tests were conducted at −20 °C.

Electrochemical testing provided further evidence of the low-temperature self-healing capability of the self-healing coatings (Figure 7). Control coatings and the self-healing coatings on steel panels were stored, scratched, and then stored inside the low-temperature chamber at −20 °C. Optical microscopy showed that the scratch width was about 20 μm. Each electrochemical cell was fabricated immediately after removing a scratched coating sample from the chamber, and the cell was maintained at −20 °C using dry ice during the test (Figure S3). The scratched control coating exhibited a current flow of 2.5 mA (Figure 7a), indicating that the damage was not healed. In contrast, the scratched STP/DD-based self-healing coating samples exhibited a very low current value of 3.3 nA (Figure 7b). The results indicate that self-healing occurred in STP/DD-based coatings at −20 °C. Repeated electrochemical tests exhibited good reproducibility.

### 3.6. Saline Solution Permeability Test

**Figure 8:**
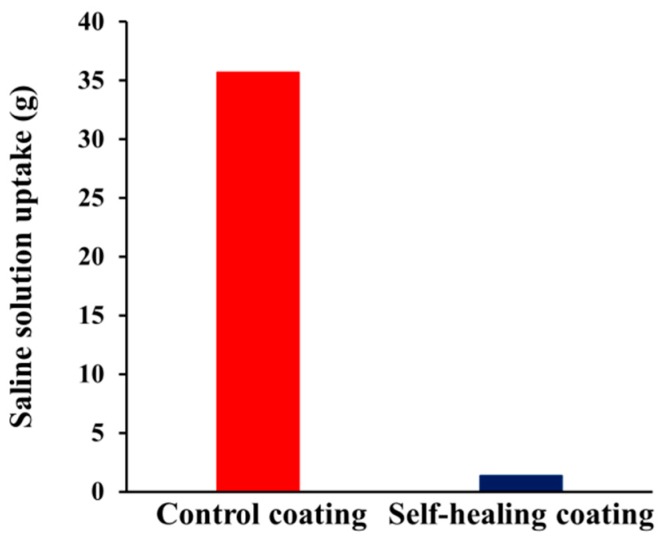
Saline solution permeability test for coated mortar specimens: graph of saline solution uptake of the specimens upon immersion of the scratched surface in a saline solution for 48 h at −20 °C.

To evaluate the applicability of our STP/DD-based self-healing coating system to cementitious composite material at low temperature, a saline solution permeability test was conducted using mortars (Figure 8). Each specimen was prepared by applying the self-healing coating formulation to one rectangular side of a square column mortar (Figure S4). Control coatings and the self-healing coatings were stored, scratched, and then stored inside the low-temperature chamber at −20 °C. Optical microscopy showed that the scratch width was about 25 μm. The scratched surface of the self-healing and control coatings was immersed in a saline solution at −20 °C for 48 h. The uptake of saline solution in the scratched control specimen was 35.7 g. In contrast, when the self-healing coating was scratched, the saline solution uptake was 1.4 g. These results indicate that successful low-temperature self-healing of damage was accomplished in our self-healing coating system by preventing saline solution permeation.

## 4. Conclusions

STP as a healing agent with DD as a catalyst was used for low-temperature self-healing. The reaction of STP in the presence of DD proceeded at low temperature (−20 °C) to generate a viscoelastic substance, which exhibited viscoelasticity even at −20 °C. STP and DD were separately microencapsulated using urea-formaldehyde resin and polyurethane as shell materials, respectively. When the self-healing coatings containing both microcapsules were damaged, STP and DD were released from the ruptured microcapsules and filled the damaged region at low temperature (−20 °C), which was confirmed by release tests. The applicability of the STP/DD-based self-healing coating system for steel panels and mortar was successfully demonstrated by corrosion tests, electrochemical tests, and saline solution permeability tests that were all conducted at −20 °C. We concluded that the STP/DD-based microcapsule-type self-healing system has good low-temperature self-healing ability. The self-healing coating has advantages because it shows effective self-healing capability under low-temperature conditions, and it can also work without any additional heating element. Our self-healing system can be applied to self-healing protective coatings exposed to cold environments.

## Figures and Tables

**Table 1:** Conversion of silanol end groups and viscosity of reaction products in the condensation reaction of STP in the presence of DD.

Reaction Temperature (°C) ^a^	Conversion (%) ^b^	Inherent Viscosity (dL/g) ^c^
	(Starting STP)	0.04
−20 (constant)	83	1.08
−20–0 (cycle)	93	1.29
−10–10 (cycle)	94	1.29
0–20 (cycle)	95	1.30

^a^ The reaction was conducted for 19 days using a STP/DD 10:1 (by mass) mixture in a mold that was 25 mm in diameter and 1 mm in height; ^b^ Measured by Fourier transform infrared spectrometry (FT-IR); ^c^ Measured at a concentration of 0.5 g/dL in toluene.

## Supplementary Materials

The following are available online at www.mdpi.com/1996-1944/10/9/1079/s1. Figure S1. FT-IR spectra of STP (a) before, and (b) after reaction at −20 °C for 19 days; Figure S2. Isothermal (25 °C) TGA thermogram of ruptured DD microcapsules; Figure S3. Schematic diagram of electrochemical test at low temperature (−20 °C); Figure S4. Procedure for saline solution permeability test: (a) coating on a mortar specimen; (b) storing the coated mortar specimen at −20 °C in a low-temperature chamber for 24 h; (c) scratching the coated mortar specimen; (d) weighing; (e) storing the specimen at −20 °C in the chamber for 12 h; (f) immersion of the coated side of the specimen in saline solution; (g) storing the specimen at −20 °C in the chamber for 48 h; (h) taking the specimen out of the saline solution and wiping the immersed surface; and (i) weighing.
