# Supplementary material for: Low-Temperature Self-Healing of a Microcapsule-Type Protective Coating
Source: Materials (Basel). 2017 Sep 14;10(9):1079. doi: 10.3390/ma10091079 (PMC5615733; doi:10.3390/ma10091079)
Supplement: Supplementary file 1 [file materials-10-01079-s001.pdf]

<Supplementary Materials>

Low-temperature self-healing of a microcapsule-type protective coating

*Dong-Min Kim, Yu-Jin Cho, Ju-Young Choi, Beom-Jun Kim, Seung-Won Jin, and Chan-Moon Chung**

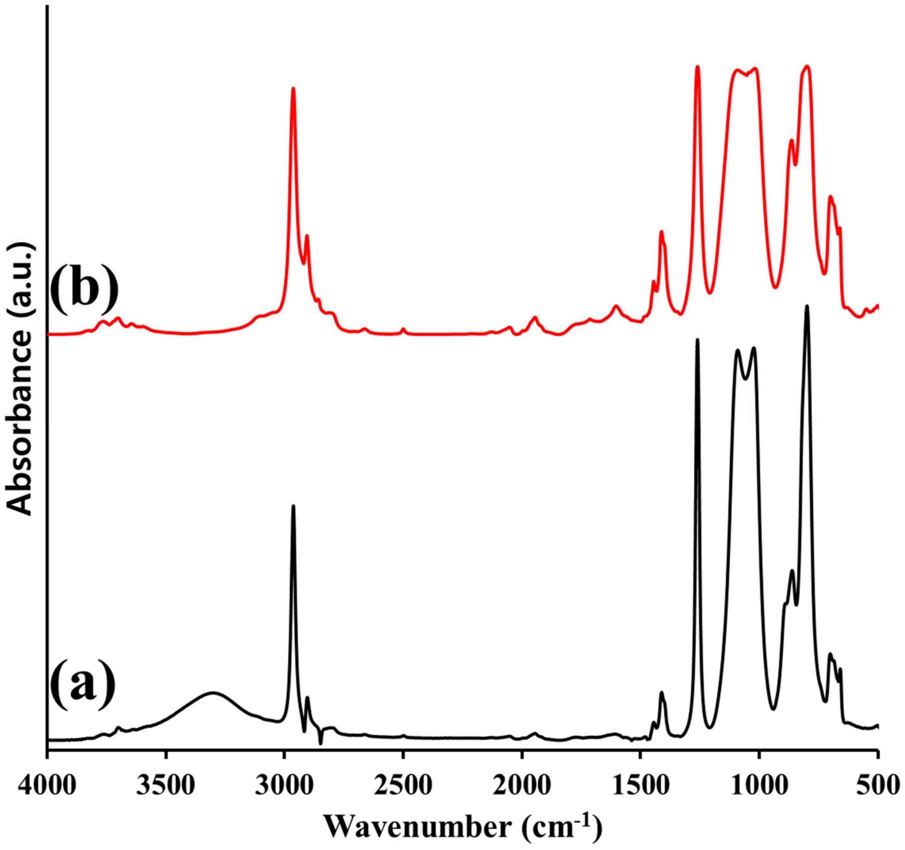

Figure S1. FT-IR spectra of STP (a) before and (b) after reaction at -20℃ for 19 days.

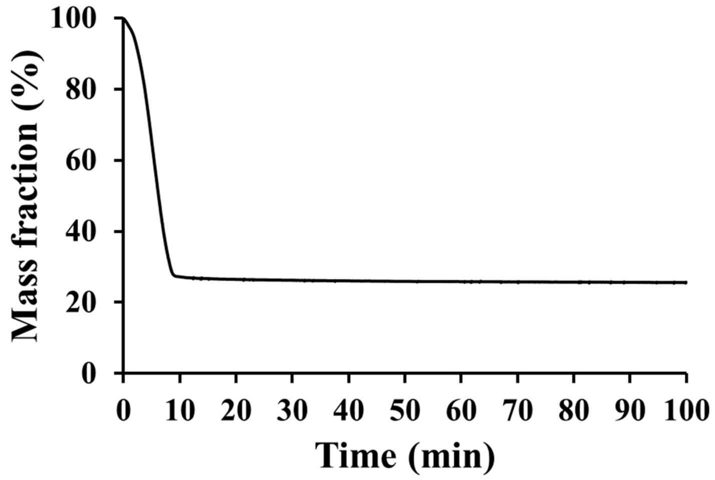

Figure S2. Isothermal (25℃) TGA thermogram of ruptured DD microcapsules.

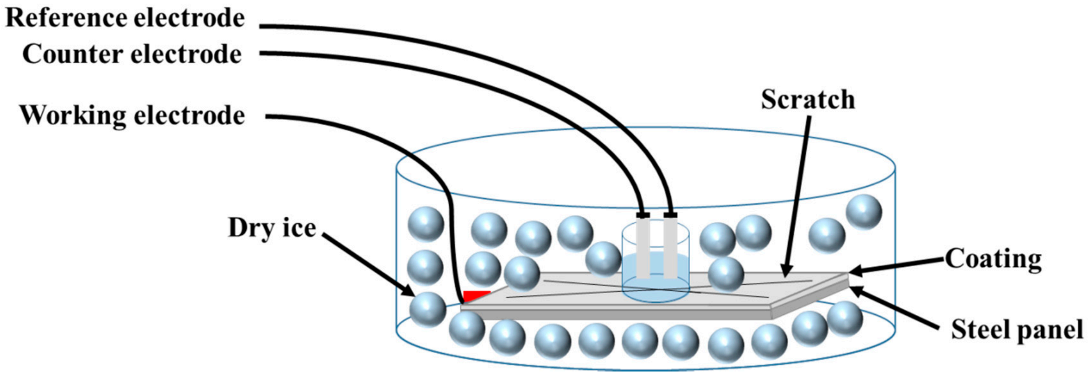

Figure S3. Schematic diagram of electrochemical test at low temperature (-20℃).

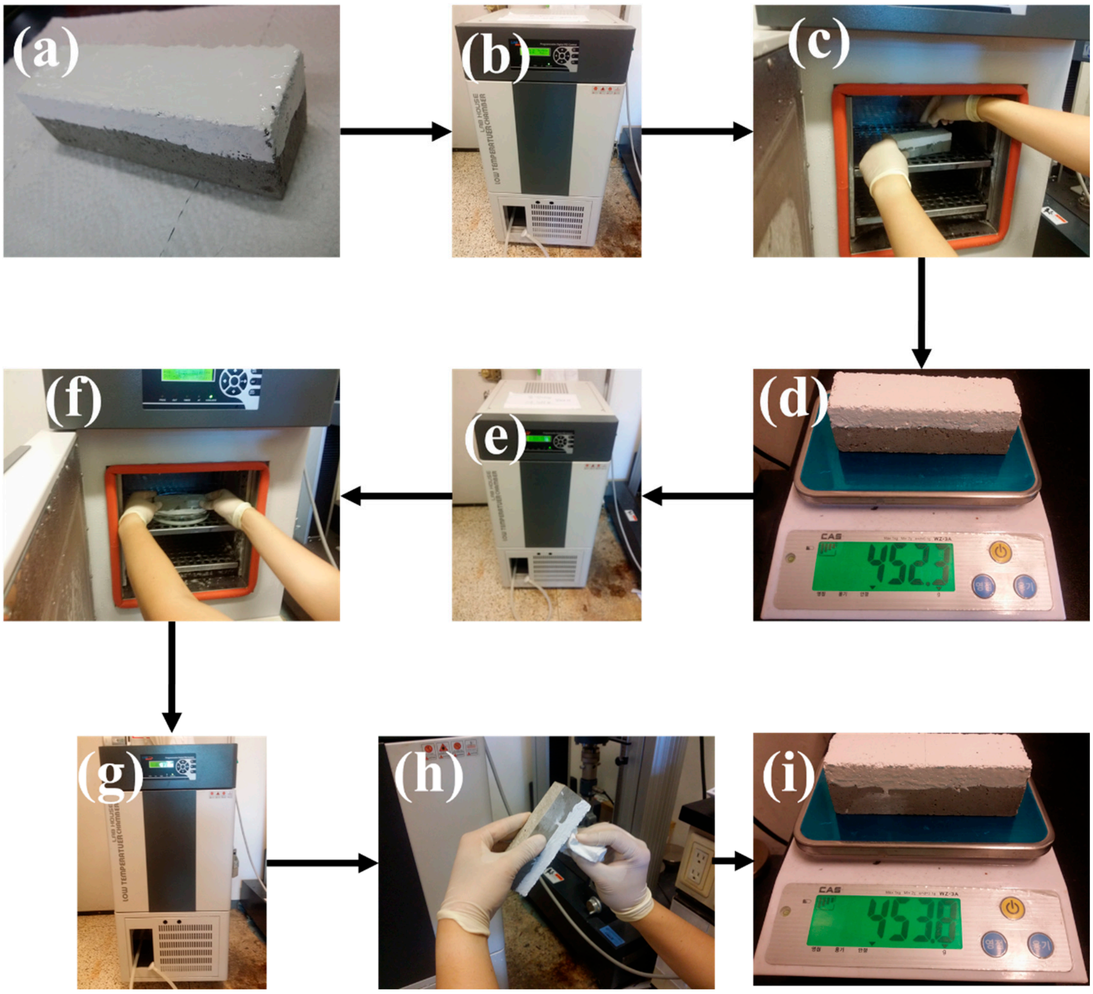

Figure S4. Procedure for saline solution permeability test: (a) coating on a mortar specimen; (b) storing the coated mortar specimen at -20℃ in a low-temperature chamber for 24 h; (c) scratching the coated mortar specimen; (d) weighing; (e) storing the specimen at -20℃ in the chamber for 12 h; (f) immersion of the coated side of the specimen in saline solution; (g) storing the specimen at -20℃ in the chamber for 48 h; (h) taking the specimen out of the saline solution and wiping the immersed surface; (i) weighing.
